# Letter to the editor “Effects of levetiracetam and lacosamide on survival and seizure control in IDH-wild type glioblastoma during temozolomide plus radiation adjuvant therapy”

**DOI:** 10.1016/j.bas.2024.102911

**Published:** 2024-08-06

**Authors:** Niher Tabassum Snigdha

**Affiliations:** Department of Dental Research, Saveetha Institute of Medical and Technical Sciences, Saveetha University, Chennai, Tamil Nadu, 600077, India

Dear Editor,

I am writing to express my appreciation for the article titled “Effects of Levetiracetam and Lacosamide on Survival and Seizure Control in IDH-wild type glioblastoma during temozolomide plus radiation adjuvant therapy” published in the recent issue of Brain and Spine (Bianconi et al., 2024) . This study aims to assess Levetiracetam and Lacosamide's potential to improve survival, seizure control, and quality of life for patients with IDH-wild type glioblastoma undergoing temozolomide plus radiation therapy. Patients' outcomes will be improved by identifying effective adjunctive treatments. Personalized and effective therapeutic strategies can be developed for managing glioblastoma based on understanding these effects.

Malignant glioblastoma (GBM) remains the most common and lethal type of tumour in the central nervous system, with just 7% 5-year survival. The prognosis for patients with GBM remains poor despite recent advances in treatment methods, such as gross total resection (GTR) and concomitant radiochemotherapy with Temozolomide (TMZ). The diagnosis of brain tumour-related epilepsy (BTRE) complicates this already challenging condition, affecting up to half of GBM patients and significantly affecting their quality of life. However, there is no consensus on the best antiseizure medication (ASM) for BTRE, while drugs like Levetiracetam (LEV) and Lacosamide (LAC), which do not interfere with chemotherapy, are widely used. This study aims to address the gap in clinical data by comparing the effectiveness of LEV and LAC in treating GBM patients regarding survival outcomes and seizure control.

The retrospective study examined the survival and seizure outcomes of GBM patients receiving TMZ chemoradiation and LEV and/or LAC. A significant difference in survival was not observed between patients using LEV or LAC, challenging previous in vitro studies suggesting potential antitumor effects of these ASMs. Further, the research revealed that LAC might be more likely to cause seizure recurrence than LEV. These findings emphasize the importance of carefully selecting ASMs based on individual patient profiles and tumour epilepsy potential. A more nuanced treatment strategy needs to consider both tumour biology and patient characteristics when managing GBM.

This study's importance lies in its potential to inform clinical practice and guide future research. This paper highlights the limitations and challenges in the current management of BTRE in GBM patients, opening the door to more targeted and personalized treatment options. Clinical trials are required to validate the antitumor effectiveness of ASMs like LEV and LAC, especially given the genetic heterogeneity of GBM. Neuro-oncologists could benefit from such research by developing treatment protocols that improve patient's quality of life and survival with GBM.

A study was undertaken to evaluate the effects of levetiracetam (LEV) and lacosamide (LAC) in patients with IDH-wild type glioblastoma (GBM) undergoing adjuvant chemoradiotherapy with temozolomide (TMZ). Despite promising in vitro data regarding the potential antitumor effects of these antiseizure medications (ASMs), neither LEV nor LAC demonstrated statistically significant differences in OS. A higher incidence rate of postoperative seizure recurrence was associated with LAC versus LEV. ASMs have the potential to be effective against specific molecular subtypes of GBM. However, there are no clinical trials to explore this potential. This study highlights the complexity of GBM treatment due to its molecular heterogeneity and emphasizes the need for further clinical trials to study this potential. This study has limitations, such as a retrospective design and limits on sample size, as well as the absence of molecular analysis of specific genes, such as p53, which may have influenced the results.

Further research should focus on molecular profiling of GBM tumours, particularly genes such as p53 and MGMT, to clarify the mechanisms of ASMs and identify patient subgroups that will benefit most from these treatments. The development of biomarker-guided treatments for GBM patients and randomized controlled trials (RCTs) comparing LEVs with LACs is essential to validate LEVs and LACs for GBM patients. Additionally, longitudinal studies are needed to measure the effectiveness of ASMs in controlling seizures and enhancing patient quality of life over time. The findings of this study can be applied to clinical practice by updating clinical guidelines, educating healthcare professionals, conducting routine molecular testing for stratification of patients, establishing collaborative research networks, and developing standardized monitoring protocols. Research relevance and acceptance depend on patient engagement through advocacy groups. This gap in research can be filled, and findings can be implemented to improve the management of BTRE in GBM patients, improving their overall survival and quality of life.

## Declaration of competing interest

The authors declare that they have no known competing financial interests or personal relationships that could have appeared to influence the work reported in this paper.
